# Interferon-α sensitizes HBx-expressing hepatocarcinoma cells to chemotherapeutic drugs through inhibition of HBx-mediated NF-κB activation

**DOI:** 10.1186/1743-422X-10-168

**Published:** 2013-05-29

**Authors:** Yanning Liu, Guohua Lou, Wei Wu, Yu Shi, Min Zheng, Zhi Chen

**Affiliations:** 1 State Key Laboratory of Infectious Disease Diagnosis and Treatment, The First Affiliated Hospital, College of Medicine, Zhejiang University, Hangzhou 310003, China

**Keywords:** HBx, Interferon-α, Hepatocellular carcinoma, NF-κB, MDR

## Abstract

**Background:**

Hepatitis B virus (HBV)-associated hepatocellular carcinoma (HCC) is characterized by high chemotherapy resistance; however, the underlying mechanism has not been fully clarified. In addition, HBx protein has been reported to play a key role in virus-mediated hepatocarcinogenesis. Therefore, the present study aims to investigate the role of HBx in the drug-resistance of HBV-related HCC and examine whether such drug-resistance can be reversed by IFN-α treatment.

**Methods:**

We established HBx-expressing cells by liposome-mediated transfection of HBx into the Huh7 cell line. MTT, Annexin V/PI, and cell cycle assay were used for determining the cellular growth inhibition, apoptosis, and growth arrest, respectively, after treatment with chemical drug. We further used tumor-bearing mice model to compare the tumor growth inhibition efficacy of ADM and 5-FU between the Huh7-HBx group and the control group, as well as the ADM + IFN-α or ADM + IMD treated group and the ADM treated group. SQ-Real time-PCR was performed to analyze the expression of MDR-associated genes and anti-apoptotic genes. Moreover, immunofluorescence and Western blotting were used to determine the subcellular localization of p65 and the phosphorylation of IκBα.

**Results:**

The IC_50_ values of Huh7-HBx cells against ADM and Amn were 2.317 and 1.828-folds higher than those of Huh7-3.1 cells, respectively. The apoptosis ratio and growth arrest was significantly lower in Huh7-HBx cells after treatment with ADM. The in vivo experiment also confirmed that the Huh7-HBx group was much more resistant to ADM or 5-FU than the control. Furthermore, the expression of MDR-associated genes, such as MDR1, MRP1, LRP1, and ABCG2, were significantly up-regulated in Huh7-HBx cells, and the NF-κB pathway was activated after HBx gene transfection in Huh7 cells. However, combined with IFN-α in ADM treatment, the HBx induced drug-resistance in Huh7-HBx cells can be partly abolished in in vitro and in vivo models. Moreover, we found that the NF-κB canonical pathway was affected by IFN-α treatment, and the expression of anti-apoptotic genes, such as Gadd45β, Survivin, and c-IAP-1 was down-regulated by IFN-α treatment in a dose-dependent manner.

**Conclusions:**

HBx protein can induce MDR of HBV-related HCC by activating the NF-κB pathway, which can be partly abolished by IFN-α treatment.

## Background

Hepatocellular carcinoma (HCC) is resistant to chemotherapeutic drugs [1], The response rates for a single cytotoxic agent are approximately 15, to 20, [2]. Moreover, only a few drugs can elicit therapeutic effect in more than 20, of patients with HCC [3]. The use of chemotherapeutic agents for advanced HCC has been very disappointing. Recently, further studies have demonstrated that the unsatisfactory effect of chemotherapy on HCC is associated with the over-expression of multi-drug resistance gene (MDR1) and consequent high levels of P-glycoprotein in HCC patients [4,5]. Furthermore, HBV-integrated HCC has developed a significantly high percentage of drug-resistance [6].

Hepatits B virus X (HBx) is a major HBV multi-functional protein that may directly or indirectly contribute to the progression of chronic hepatitis B to HCC [7]. The over-expression of HBx protein can induce cell transformation [8]. In addition, HBx protein can interact with p53 tumor suppressor gene, and inactivate this “gene guard” [9]. HBx protein has been confirmed to contribute to NF-κB signaling pathway activation [10,11]. NF-κB has a central role in the regulation of diverse biological processes, including immune response, development, cell growth, and survival [12,13]. However, continuous activation of NF-κB leads to carcinogenesis and tumor development [14].

Current anti-tumor treatments, such as radiotherapy, chemotherapy, immunotherapy, and suicide gene therapy, act on tumor by inducing tumor cell apoptosis [15,16]. Evidence have shown that activated NF-κB can induce the expression of anti-apoptotic genes, and that the consistent activation of NF-κB is probably involved in tumor drug-resistance development [17,18]. As a result, we hypothesized that NF-κB mediates the up-regulation of anti-apoptotic gene expression, which is induced by HBx protein that contributes to the development of the drug-resistance of HBV-integrated HCC.

Generally, new drugs are needed to solve the problem of drug-resistance in chemotherapy. However, this expectation is a little unrealistic when the long drug-development period and high failure ratio is considered. Thus, it seems more reasonable to select an “adjuvant drug” that can increase the sensitivity of tumor cells to chemotherapeutic drugs. Adjuvant drug can improve the anti-tumor effect of chemotherapeutic drugs and reduce the drug dose and toxicity.

Interferon-α (IFN-α) has been discovered based on its antiviral activity. IFN-α is one of the drugs first approved by FDA for the treatment of chronic HBV infection, and has been clinically applied for 20 years [19]. IFN-α exerts its antiviral effect by inducing the expression of some protective host proteins such as PKR and MyD88. Recent studies have shown that IFN-α can accelerate TNF-induced tumor cell apoptosis by up-regulating Fas expression [20], while other studies have shown that pretreatment with IFN-α can inhibit the TRAIL-mediated NF-κB activation, thereby increasing the response of hepatoma cells to TRAIL-induced apoptotic signal [21]. Based on these facts, we examined whether IFN-α can improve chemo-sensitivity in tumor cells by inhibiting the HBx protein-induced activation of the NF-κB signaling pathway and whether IFN-α can contribute to the reversion of tumor drug-resistance. Moreover, we hypothesize that IFN-α may be a candidate adjuvant drug for HBV-integrated HCC chemotherapy.

## Results

### Introduction of HBx contributed to the drug-resistance development of Huh7 cells

#### Transfection of HBx reduces the chemosensitivity of Huh7 cells

**Figure 1:**
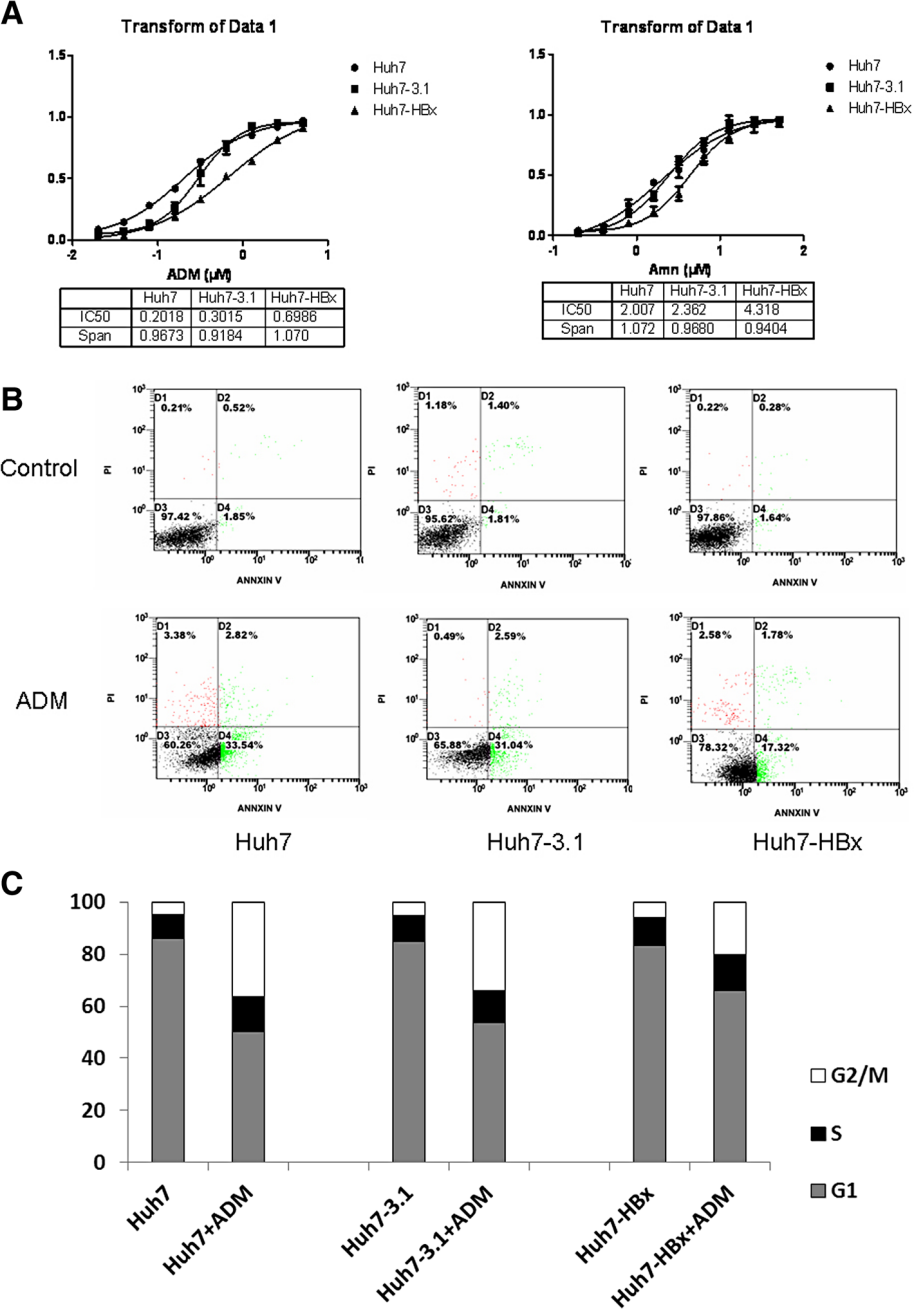
**Increase in drugs resistance induced by HBx. A**) The IC_50 _value of ADM and Amn against Huh7, Huh7-3.1, and Huh7-HBx cells respectively. Huh7-HBx cells represent a higher resistance of 2.317 and 1.828 times, respectively, than Huh7-3.1 cells against these two chemotherapeutic drugs. **B**) After treatment with ADM for 24 h, the Annexin V/PI assay was used for the analysis of cell apoptosis. A significantly less degree of apoptosis was found in Huh7-HBx cell than in Huh7-3.1 cell (*P* < 0.05). **C**) Cell cycle analysis of Huh7, Huh7-3.1 and Huh7-HBx cells after 24 h of treatment with ADM, as determined by flow cytometry for DNA content.

To determine the impact of HBx protein on the drug-sensitivity of hepatoma cells, we constructed a pcDNA3.1-HBx plasmid, an expression vector of HBx protein, and then transfected it into Huh7 cells. After verifying the expression of HBx in Huh7-HBx cells (data not show), we performed MTT experiment, and then ayalyzed by GraphPad Prism 5 software to evaluate the destructive effect of ADM and Amn on Huh7, Huh7-3.1, and Huh7-HBx cells, respectively. The IC_50_ of ADM and Amn in Huh7-HBx cells were 2.317 and 1.828 times of those in Huh7-3.1 cells (*P*<0.01), as shown in Figure 1A. We further assessed the apoptosis ratio in Huh7, Huh7-3.1, and Huh7-HBx cells by Annexin V/PI analysis after treatment with 1 μg/ml ADM for 24 h. We found a significantly lower apoptosis ratio in Huh7-HBx (15.78 ± 3.54,), compared with Huh7 (31.09 ± 4.54,) and Huh7-3.1 cells (30.15 ± 4.41,) (Figure 1B). In addition, the ADM-induced G2/M growth arrest in Huh7-HBx cells were significantly lower compared with that in Huh7 and Huh7-3.1 cells (Figure 1C).

#### Transfection of HBx reduces the chemosensitivity of Huh7 in xenograft mice model

Nude mice were inoculated s.c. into the right armpit with Huh7, Huh7-HBx, or Huh7-3.1 cells. After three weeks, 5-FU (25 mg/kg × d), ADM (2 mg/kg × d), or normal saline was administered by i.p. injection. Table 1 shows that the administration of 5-FU in the Huh7 and Huh7-3.1 hepatoma models for 14 days reduced the tumor growth by 74.20, and 71.18,, respectively, and the administration of ADM reduced the tumor growth by 68.36, and 69.10,, respectively. Meanwhile, the i.p. administration of 5-FU or ADM in the Huh7-HBx hepatoma models for 14 days caused much less reduction in tumor growth, with only 39.56, and 42.26, respectively (*P*<0.01).

**Table 1:** HBx reduces the chemosensitivity of Huh7 in xenograft mice model

**Tumor**	**Treatment (n = 5)**	**Dosage (mg/kg × d)**	**Tumor weight (g)**	**Growth inhibition (%)**	**P, *t*-Test**
Huh7	Vehicle	0	2.089 ± 0.229	-	-
Huh7-3.1	Vehicle	0	2.11 ± 0.259	-	p>0.05
Huh7-HBx	Vehicle	0	2.068 ± 0.195	-	p>0.05
Huh7	5-FU	25	0.528 ± 0.19	74.72	-
Huh7-3.1	5-FU	25	0.608 ± 0.253	71.18	p>0.05
Huh7-HBx	5-FU	25	1.25 ± 0.307	39.56	p<0.01
Huh7	ADM	2	0.661 ± 0.218	68.36	-
Huh7-3.1	ADM	2	0.652 ± 0.162	69.1	p>0.05
Huh7-HBx	ADM	2	1.194 ± 0.211	42.26	p<0.01

Student’s *t *test was used to assess the significance of tumor growth inhibition of each group compared with vehicle (normal saline -treated group).

These data indicated the direct role of HBx protein in regulating the drug-resistance of hepatoma cells.

### HBx-induced drug resistance are associated with NF-κB pathway activation

#### HBx induces NF-κB pathway activation

**Figure 2:**
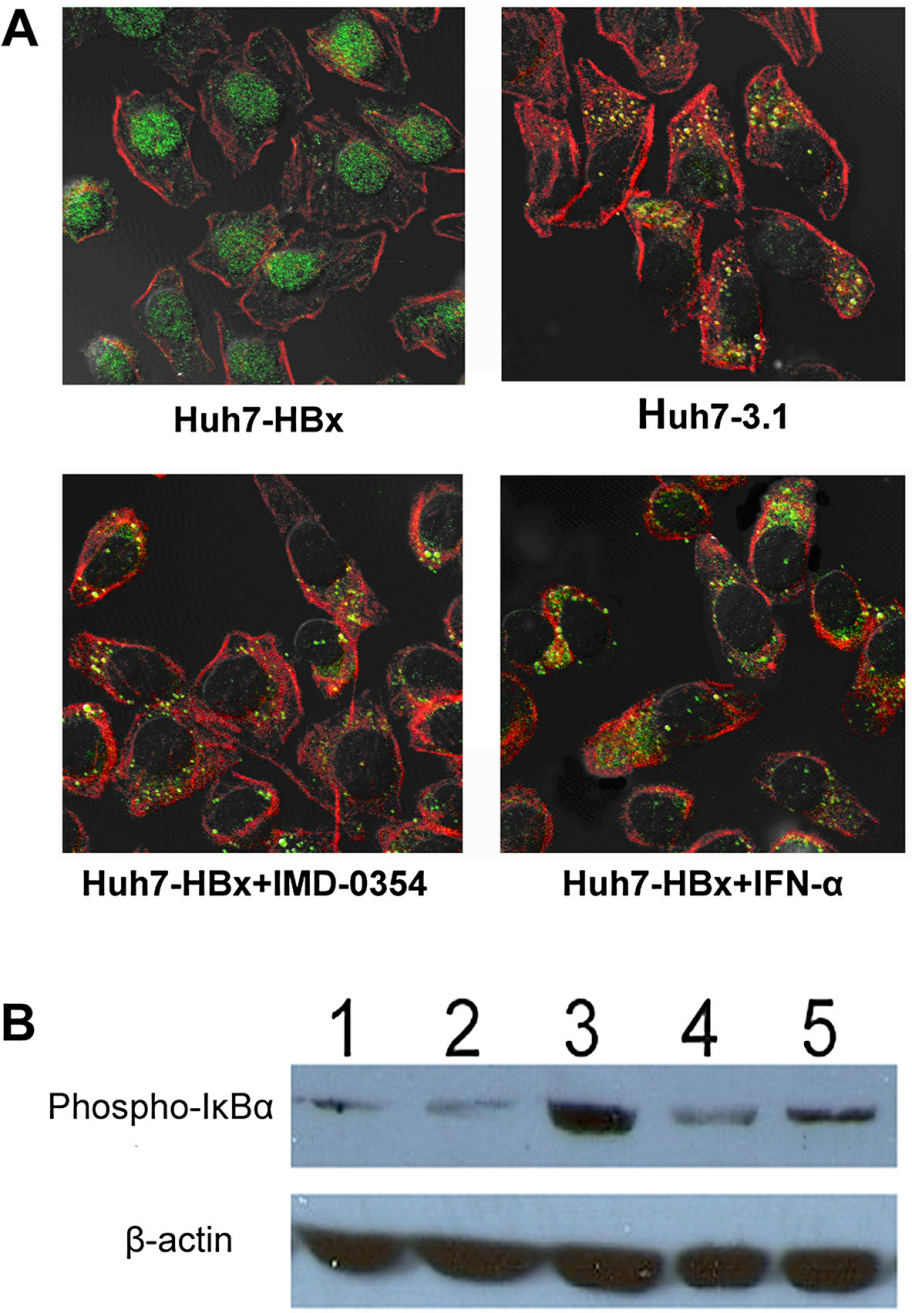
**HBx induces NF-κB pathway activation. A**) Confocal immunofluorescence analysis of Huh7-3.1, Huh7-HBx, and Huh7-HBx treated with IMD-0354 or IFN-α, using p65 antibody (green). Actin filaments have been labeled with Alexa Fluor 555 phalloidin (red). P65 is mainly localized in the cytoplasm of Huh7-3.1 cells, while was partly translocated into the nucleus of Huh7-HBx cells. However, the level of p65 in the nucleus of Huh7-HBx cells decreased to some extent after treatment with IMD-0354 or IFN-α. **B**) The expression level of phosphorylated IκBα was detected by Western blotting (lane 1, Huh7 cells; lane 2, Huh7-3.1 cells; lane 3, Huh7-HBx; lane 4, Huh7-HBx treated with IMD-0354; lane 5, Huh7-HBx treated with IFN-α).

Activated NF-κB1/p65 would translocate from the cytoplasm to the nucleus, hence, we evaluated NF-κB activation by immunofluorescence staining of p65, and then compared cytoplasmic and nuclear localization of p65 by confocal. Figure 2A shows that p65 was mainly localized in the cytoplasm of the Huh7-3.1 cells, while a considerable percentage of p65 was found in the nucleus of Huh7-HBx cells. However, after incubating the Huh7-HBx cells with an IKKβ specific inhibitor, IMD-0354, for 24 hours, the level of p65 in the nucleus decreased to some extent.

Subsequently, we determined the phosphorylation of IκBα, a cytoplasmic inhibitory molecule of NF-κB. Western blot analysis revealed the presence of phosphorylated IκBα in Huh7-HBx cells, while pretreated with IMD-0354 for 24 h decreased the level of phosphorylated IκBα in Huh7-HBx cells significantly (Figure 2B).

#### HBx-induced drug resistance can be abolished by inhibiting the NF-κB pathway

**Figure 3:**
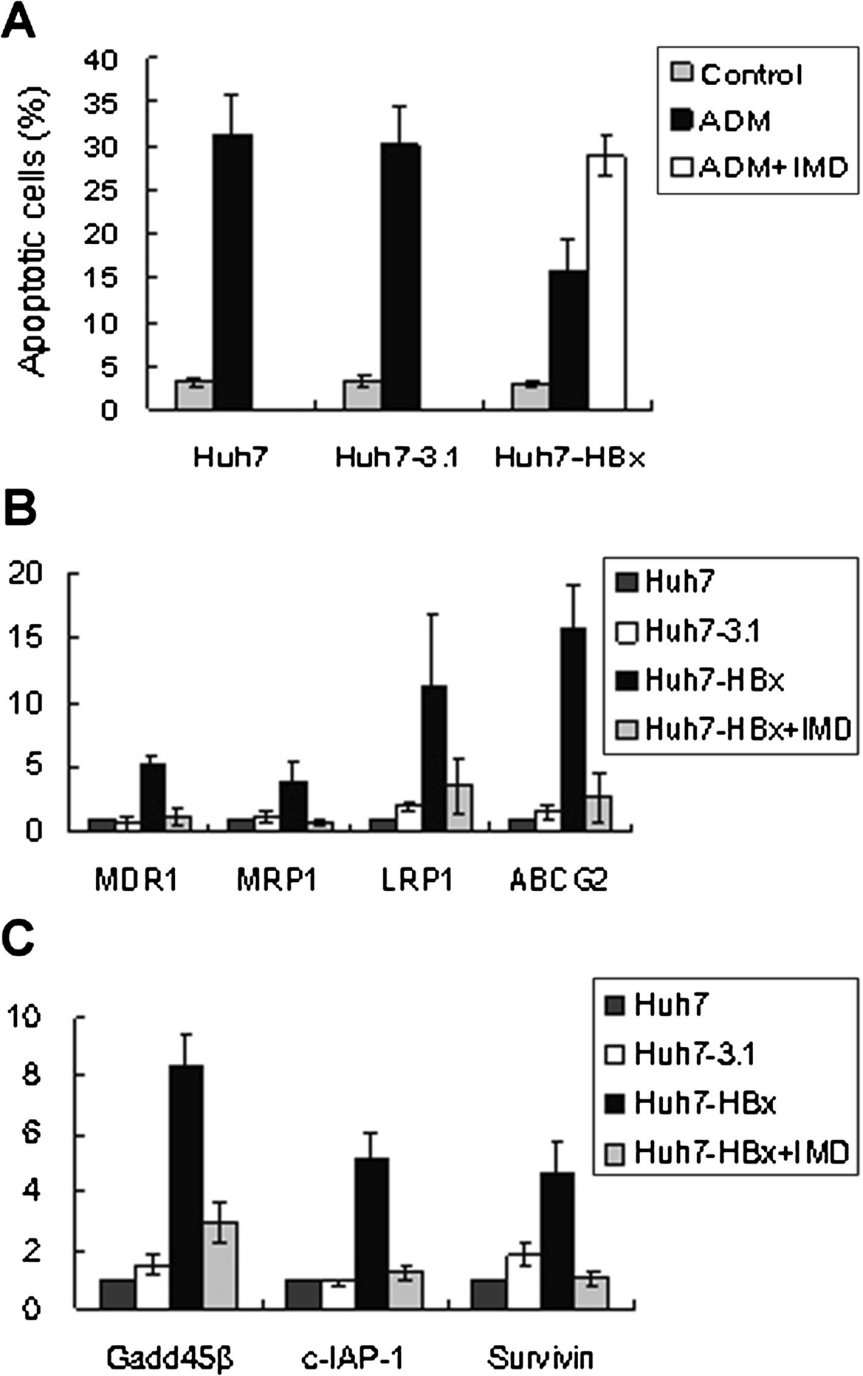
**HBx up-regulated the expression of MDR-associated genes and anti-apoptosis genes mediated by the NF-κB pathway. A**) Apoptosis index of Huh7, Huh7-3.1, and Huh7-HBx cells after treatment with ADM. The apoptosis ratio in Huh7-HBx cells was significantly lower than that of the Huh7-3.1 cells (*P* < 0.01). However the level of apoptosis index in the Huh7-HBx cells increased to some extent after co-treatment with the NF-κB pathway inhibitor, IMD-0354 (*P*<0.05). **B**) The expression of MDR associated genes of Huh7, Huh7-3.1 and Huh7-HBx cells was detected by real-time RT-PCR, with β-actin as internal control. The gene expression levels of Huh7 cells were set as calibrator to compare with others. **C**) The transcript level of anti-apoptosis genes in Huh7, Huh7-3.1, and Huh7-HBx cells was detected by real-time RT-PCR analysis with β-actin as internal control. Logarithmic scale expression values were normalized to Huh7 (ref =1, n = 3).

We further assessed whether the HBx-induced drug resistance are affected by blocking the NF-κB pathway. Annexin V-FITC/PI analysis showed that the apoptosis ratio of Huh7-HBx cells after incubated with IMD-0354(2 μM) increased to some extent (Figure 3A).

Recent studies indicated that the transactivating forms of NF-κB (e.g., dimers containing the p65/RelA subunit) may up-regulate the expression of the *MDR1/P-gp* gene, which is involved in drug resistance. We also assessed whether HBx-induced drug resistance are associated with the up-regulation of MDR-associated genes, such as MDR1, MRP1, LRP1, and ABCG2. As shown in Figure 3B, the expression of these genes dramatically increased in Huh7-HBx, but maintained at a stable level in Huh7-3.1 cells. Huh7 cells were set as calibrator for comparison with others. However, the expression of MDR-associated genes dramatically decreased after incubating the Huh7-HBx cells with 2 μM IMD-0354 for 24 h (Figure 3B).

Cancer cells would activate the NF-κB pathway to up-regulate the expression of anti-apoptotic genes, such as c-IAP-1, c-IAP-2, Bcl-Xl, Gadd45β, and Survivin, to avoid apoptosis. We further assessed whether the HBx-induced drug resistance are associated with the up-regulation of the expression of these anti-apoptotic genes. We found a significant up-regulation of Gadd45β, Survivin, and c-IAP-1 level in Huh7-HBx cells, compared with that in Huh7-3.1 cells. However, the expression of these genes dramatically decreased after incubating the Huh7-HBx cells with 2 μM IMD-0354 for 24 h (Figure 3C).

These results suggested that HBx-induced drug resistance are mediated by the NF-κB pathway, and this drug resistance can partly be abolished by inhibiting NF-κB activation through IMD-0354 treatment.

### Interferon-α sensitizes HBx-expressing hepatoma cells to ADM by inhibiting the HBx-mediated NF-κB activation

**Figure 4:**
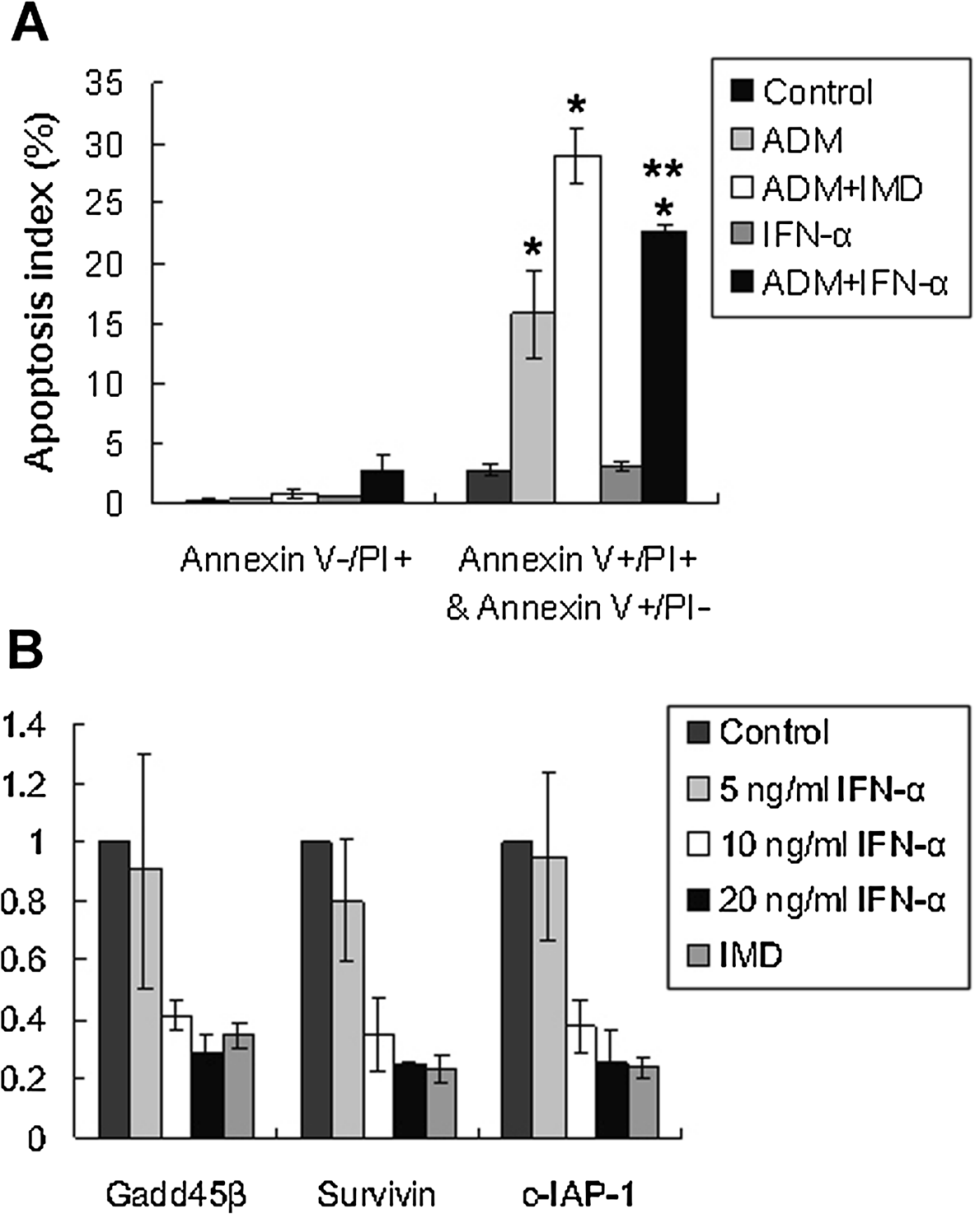
**Interferon-α diminishes Huh7-HBx cells resistance to ADM. A**) Apoptotic index quantitated by FACS. Compared with Huh7-HBx cells treated with either IFN-α or ADM, the cells treated with both IFN-α and ADM clearly showed an increase in annexin^+^/PI^- ^& annexin^+^PI^+ ^cell population. Data are showed as mean ± SD, and statistically analyzed with Student’s *t *test assay. * *P* < 0.05, ** *P* < 0.01, each cells *v.s. *controle (non-treated Huh7-HBx cells)**. B**) The transcript level of the anti-apoptosis genes in Huh7-HBx cells (control) and IFN-α or IMD-0354 pretreated Huh7-HBx cells were detected by real-time RT-PCR analysis, with β-actin as internal control. Logarithmic scale expression values were normalized to Huh7-HBx (ref =1, n = 3).

Confocal and Western blot analysis showed that IFN-α decreased the NF-κB activity in HBx-producing cells (Figure 2A, 2B). Based on this result, the inhibition of NF-κB activity by IFN-α was expected to decrease the resistance to ADM. Therefore, we analyzed the viability and apoptosis in Huh7-HBx cells, which have high NF-κB activity, by treating these cells with ADM and IFN-α. Compared with Huh7-HBx cells treated with either IFN-α or ADM, the cells treated with both IFN-α and ADM clearly showed an increase in annexin V binding of the cell population (Figure 4A).

The tumor growth assay in nude mice also showed that IFN-α can sensitize HBx-expressing hepatoma cells to ADM treatment. The weight of the neoplasms from ADM + IFN-α treated mice were significantly smaller than the tumors of the Huh7-HBx implanted mice (P < 0.05). In addition, daily administration with 5 mg/kg of IMD-0354 (NF-κB inhibitor) combined with ADM also significantly suppressed tumor expansion in Huh7-HBx bearing nude mice compared with ADM only (Table 2).

**Table 2:** IFN-α improve the chemosensitivity of Huh7-HBx xenograft mice model to ADM treatment

**Treatment (n = 5)**	**Tumor weight (g)**	**Growth inhibition (%)**	**P, *t*-Test**
Vehicle	2.102 ± 0.165	-	-, -
ADM	1.31 ± 0.159	37.69	p<0.01, -
IFN-α	1.75 ± 0.135	16.73	p<0.05, -
ADM + IFN-α	0.725 ± 0.098	65.49	p<0.01, *p<0.01
ADM + IMD	0.622 ± 0.095	70.41	p<0.01, *p<0.01

Student’s *t *test was used to assess the significance of tumor growth inhibition between each group. Left “p”: each group compared with vehicle (saline treated Huh7-HBx bearing group). Right “*p”: ADM + IFN-α or ADM + IMD treated group compared with ADM treated group.

The real time-PCR results for Gadd45β, Survivin, and c-IAP-1 showed that the expression of these anti-apoptotic genes was dose-dependently repressed by IFN-α treatment in Huh7-HBx cells (Figure 4B).

These results suggested that IFN-α-mediated drug resistance disruption may be associated with the down-regulation of anti-apoptotic gene expression by inhibiting NF-κB activation.

## Discussion

Chronic infection of hepatitis B and C can advances to cirrhosis and HCC. Recent studies have reported that HepG2.2.15 cells (HBV-producing cells) exhibit higher resistance to 5-FU than HepG2 cells, indicating a close relationship between HBV infection and HCC drug resistance [6]. However, the underlying mechanism remains unclear. HBx gene, the smallest open reading frame in viral DNA, encodes a 154-amino acid length protein. HBx functions in a variety of signaling pathways including the NF-κB-related pathways; hence, may have an important role in HBV-related tumorigenesis and tumor progression [22,23]. The present study aims to investigate the role of HBx in HBV-induced drug resistance of HCC, and examine whether such drug-resistance can be reversed by IFN-α treatment.

We first used in vitro HBx-expression hepatoma cell lines to analyze the effect of HBx gene induction, and found that Huh7-HBx cells have increased drug resistance than Huh7-3.1 cells. Our data showed that the IC_50_ values of Huh7-HBx cells against ADM and Amn were 2.317 and 1.828 -folds higher than those of Huh7-3.1 cells, respectively. When treated with 1 μg/ml ADM, Huh7-HBx cells exhibited significantly lower apoptosis rate and G2/M growth arrest than Huh7-3.1 cells. We also used HepG2 and SMMC-7721(SMMC) hepatoma cell lines, along with Huh-7 cells, to investigate the role of HBx in the HBV-induced drug resistance of HCC. After transfection with pcDNA3.1-HBx, HepG2-HBx and SMMC-HBx also exhibited increased drug resistance than the pcDNA3.1 vector transfected cells (data not shown). We further used a HCC murine model to confirm the HBx-induced drug resistance. The administration of 5-FU and ADM reduced the tumor growth in the Huh7-3.1 group by approximately 70, while the Huh7-HBx group exhibited much less reduction in tumor growth, with only about 40, (*P*<0.01). All these results show that HBx is closely related to the HBV-induced drug resistance of HCC.

NF-κB is not a single protein, but a collection of dimeric transcription factors composed of members of the Rel family with five closely related DNA binding proteins: RelA (p65), RelB, c-Rel, NF-κB1/p50, and NF-κB2/p52. In resting cells, NF-κB dimers are sequestered in the cytoplasm as latent complexes through binding to the members of a family of ankyrin repeat domain (ARD)-containing inhibitors called IκB (inhibitor of κB) proteins, which interact with the RHD of NF-κB proteins [24]. There are two distinct NF-κB activation pathways: canonical (or traditional) and non-canonical (or alternative) pathways. Our results have demonstrated that the HBx-induced drug resistance of HCC is associated with the activation of NF-κB canonical pathways, which was based on inducible IκBα degradation, allowing NF-κB dimers (mainly p65/p50 dimers) to accumulate in the nucleus and activate transcription. The presence of spontaneously phosphorylated IκBα and activated NF-κB in the Huh7-HBx cells was observed through immunofluorescence and Western blotting analysis. Further blocking test with selective IKKβ inhibitor, IMD-0354 (blocks IκBα phosphorylation and prevents the induction of p65 nuclear translocation), which against NF-κB canonical pathway indicated a partial reversion of drug resistance of Huh7-HBx. After treatment with IMD-0354 for 24 h, Huh7-HBx exhibited increased apoptosis in response to ADM and 5-FU. Moreover, the HBx-induced up-regulation of drug resistance-associated genes (such as MDR1, MRP1, LRP1, and ABCG2) and anti-apoptotic genes, such as Gadd45β, Survivin, and c-IAP-1, was repressed. Moreover, our data indicated that HBx may induce HCC drug resistance by activating the NF-κB canonical pathways. Hung et al. also demonstrated that HBV pre-S2Δ (HBV pre-S2 mutant large surface) protein can induce resistance to 5-FU treatment in Huh-7 cells through the induction of NF-κB p65 [25]. However, another study showed that HBV causes drug resistance through the non-canonical (alternative) pathway, in which NIK phosphorylates ΙΚΚα, thereby activating NF-κB [6]. Thus, we hypothesized that other HBV proteins may contribute to progression of HCC drug resistance by activating the NF-κB alternative pathways in an IKKβ-independent way.

Several drugs, including ADM and docetaxel, have been developed for treating breast, prostate, and lung cancers with great success. However, their anticancer effects are diminished because of their intrinsic or acquired drug resistance, which involves the over-expression of P-glycoprotein (P-gp) or multidrug resistance protein 7 (MRP7) [26]. Several strategies have been studied to overcome MDR mechanism including the use of novel drug delivery systems, co-administration of P-gp inhibitors, and the development of novel anticancer drugs that can circumvent MDR. Scientists have also successfully discovered several new compounds to overcome MDR. Ixabepilone, for example, has been approved by the FDA as a drug for MDR breast cancer [27]. However, an effective drug against MDR HCC has not been found yet. Thus, selecting “adjuvant drug” that can increase the sensitivity of hepatoma cells to existing drugs will contribute significantly to the progress of cancer chemotherapy.

IFN-α has an essential role in antiviral host defenses and has been used clinically for the treatment of chronic HBV infection [28]. IFN-α cannot kill the viruses directly; however, it functions by activating the transcription factor of antiviral proteins and inducing the synthesis of these proteins. These antiviral proteins, such as PKR (double strand RNA activated protein kinase), inhibit virus replication by inhibiting protein synthesis by phosphorylate the translation initiation factor eIF-2 [29]. Moreover, PKR can also interact with IKKβ and interfere with the NF-κB signaling pathways [30].

We found that IFN-α treatment improved the drug sensitivity of HBx-expressing hepatoma cells. The Huh7-HBx cells in the IFN-α treated group exhibited a remarkably higher apoptosis in response to 5-FU and ADM than the untreated group. The tumor growth assay in nude mice also showed that IFN-α can sensitize Huh7-HBx cells to ADM treatment. The weight of the neoplasms from ADM + IFN-α treated mice were significant lower than the tumors from ADM treated Huh7-HBx-bearing mice (P < 0.05). In addition, daily administration with 5 mg/kg of IMD-0354 (NF-κB inhibitor) combined with ADM also significantly suppressed tumor expansion in Huh7-HBx-bearing nude mice compared with ADM only. Moreover, the up-regulation of anti-apoptotic gene expression was largely inhibited by IFN-α treatment. Our study showed that IFN-α treatment significantly reduced the nuclear concentration of p65 and the level of phosphorylated IκBα in Huh7-HBx cells, which was consistent with the effect of IMD-0354 pretreatment. These results indicated that IFN-α may inhibit one essential step in NF-κB canonical pathway, and thus reduced the HBx-induced NF-κB activation. However, the specific step of IFN-α interfering with the NF-κB canonical pathway is still unknown. Thus, further investigation is needed to verify whether IFN-α acts directly on IKKβ as IMD-0354 or suppress the degradation of IκBα, IκBβ, and p105.

In conclusion, we indicated that HBx induced drug resistance was associated with NF-κB canonical pathway activation. We also demonstrated that IFN-α can inhibit the HBx-mediated activation of NF-κB. These results suggest that IFN-α treatment may be a useful strategy to enhance the response to chemotherapy in HBV-integrated HCC through inhibiting the NF-κB activation triggered by HBx.

## Materials and methods

### Cell lines

Huh7 cells were cultured in Dulbecco’s Modified Eagle’s Medium (Gibco) supplemented with 10, fetal bovine serum (FBS, Gibco). The stable transfected Huh7-HBx and Huh7-3.1 cells were cultured in DMEM supplemented with 10, FBS and 800 μg/ml G418.

### The construction of pcDNA3.1-HBx plasmids and stable transfection

The expression vector pcDNA3.1-HBx was constructed by inserting HBx DNA fragments into pcDNA3.1 vector. The primers were: HBx-up: 5′-acttaagcttgccaccatggctgctaggctg-3′, HBx-down:5′- tagactcgagttacagatcctcttctgagatgagtttttgttcggcagaggtgaaaaagttg-3′. After by PCR amplification using DNA sample of HepG2.2.15 cells, which is an HBV cell model by transducing of HBVayw genotype into HepG2 cells, the HBx DNA fragment was inserted between the *Hin*d III and *Xho* cloning sites of the pcDNA3.1 vector. And after amplification in *DH-5*α, we used the plasmid DNA mini kit (Simgen) to purify the plasmids. Huh7 cells at 70-80, confluence were transfected with plasmids, using lipofectamine 2000 (Life Technologies, Inc.). Plasmid transfections were performed according to protocols supplied with the reagents. At 48 h post-transfection, cells were cultured in the presence of 1.5 mg/ml G418. After 21 ~ 2cbu in selective medium, individual G418-resistant colonies were isolated. Expression of HBx in Huh7-HBx cell lines was verified by RT-PCR and Western Blot. The control cell line (Huh7-3.1) was generated from cells transfected with the vector alone and selected using G418. After isolation of resistant clones, the concentration of G418 was changed to 800 μg/mL.

### MTT assay

MTT is a colorimetric technique for detecting the reduction of MTT (Methyl thiazolyl tetrazolium, Sigma) by mitochondrial dehydrogenase to blue formazan product, which reflects the normal function of mitochondria and hence the measurement of cytotoxicity cell and viability. The cells were seeded onto 96-well plates and treated with ADM (doxorubicin) or Amn (amonafide) (Sigma) for 72 h. The medium was removed, and the cells were incubated with a solution containing 0.5 mg MTT/mL phosphate-buffered saline at 37°C for 4 h. The MTT solution was removed and the cells were overlaid with 100 μL/well DMSO for 15 min at 37°C. The OD value was measured using a Bio-Rad microplate reader at 570 nm with DMSO as blank. Triplicate wells were assayed for each condition. Percent growth inhibition of cells exposed to treatments was calculated as follows:, Inhibition = 100 - (Test OD/Non-treated OD) × 100. The data were then analysesed by GraphPad Prism 5 software to get IC_50_ value.

### Cell apoptosis and cell cycle analysis by FACS

Cell apoptosis analysis: Huh7, Huh7-HBx and Huh7-3.1cells were plated and grown overnight until they reach 80, confluence, then the cells were treated with 1 μg/ml ADM or/and IMD-0354. Subsequently, detached cells in the medium were collected, and the remaining adherent cells were released by trypsinization. The cells were washed with phosphate-buffered saline (PBS) and resuspended in 250 μL binding buffer (annexinV-FITC kit; BECKMAN COULTER) containing 5 μL of annexin V-FITC stock and 10 μL of 20 μg/mL propidium iodide (PI). After incubated for 10 min at room temperature in a light protected area, the samples were analyzed by FACS (BECKMAN COULTER FC500 MPL) using muticycle software. We could discriminate intact cells (annexin^-^/PI^-^) from apoptotic cells (annexin^+^/PI^-^ & annexin^+^PI^+^) and necrotic cells (annexin^-^/PI^+^) after treatment with ADM.

Cell cycle analysis: Cell cycle analysis was performed by propidium iodide staining (Sigma, St. Louis, MO). Cells were fixed in 70, ethanol, incubated with 0.1, RNase A in PBS at 37 C for 30 min and resuspended in PBS containing 25 μg/mL propidium iodide (PI) for 30 min at room temperature. The stained cells were analyzed for DNA content by FACS (BECKMAN COULTER FC500 MPL) using muticycle software.

### Tumor growth assays in nude mice

In vivo seven-week-old specific pathogen-free male Nude mice (weight, 18 ~ 22 g) were supplied by Zhejiang Academy of Medical Science. And the study was approved by the animal ethics committee of Zhejiang University (approval ID, Zju2009-1-01-025Y). Mice were inoculated s.c. into the right armpit with Huh7, Huh7-HBx or Huh7-3.1cells. After 3 weeks, 5-fluorouracil (25 mg/kg), ADM (2 mg/kg) or/and IFN-α (4 MIU/kg × d), IMD-0354 (5 mg/kg) was administered by i.p. injection once a day for 14 days. Saline was injected in nude mice as a control. The inhibition rate was calculated as [(average tumor weight of normal saline group - average tumor weight of test group)/average tumor weight of normal saline group] × 100.

### Immunofluorescence and confocal analysis

Cells were attached to the gelatin-coated glass coverslip overnight. Then the coverslips were washed with PBS three times and then fixed with 4, paraformaldehyde for 10 min at 4°C. After treated with 0.3, Triton X-100 for 10 min, then washed with PBS again, and incubated with blocking buffer (1, BSA in PBS) for 30 min to minimize non-specific adsorption of the antibodies to the coverslip. Then incubated with primary antibodies against NF-κB p65 (1:100, cell signaling) at 4°C overnight. The coverslips were then washed in PBS and incubated with FITC-coupled secondary antibodies (1:200, Santa Cruz) for 30 min at room temperature. And then incubated with Alexa Fluor 555 phalloidin for 20 min at room temperature. After washed in PBS,the cell coverslips were mount with glycerol and examined by confocal (Olympus).

### Western blot analysis

To determine the levels of protein expression, whole-cell extracts were prepared and fractionated using SDS-PAGE. After electrophoresis, the proteins were electrotransferred onto nitrocellulose membranes, blotted with phosphorylated IκBα antibody, and detected using an enhanced chemiluminescence regent (Amersham). Blots were visualized by ECL-associated fluorography (Pierce Inc.).

### Semi-quantitative- real time PCR (SQ-real time-PCR)

The cells were collected by trypsinization, and then the total RNA was extracted using TRIzol Reagent (Invitrogen, USA) according to the manufacturer’s protocol. The cDNA was produced by RT using Reverse transcription kit (Takara). Then the products were used for analysis on an ABI PRISM 7900 Sequence Detection System using SYBR^®^ Premix Ex Taq^TM^ kit (Takara). The expression of the following genes was evaluated: MRP-1, LRP, MDR-1, ABCG2, Gadd45β, Survivin and c-IAP-1. β-actin was used as the endogenous control to normalize all the above target genes; to determine the relative quantification, the comparative threshold cycle (Ct) method was used. The gene expression levels of Huh7 cells or Huh7-HBx cells were set as calibrator to compare with others. All Assays-on-Demand™ Gene Expression Products consisted of target assays and endogenous control assays to amplify and detect expression of specific RNA sequences.

### Statistical analyses

Data are expressed as mean ± SEM; all values were calculated, graphed and analyzed statistically using Student’s *t*-test. *P* < 0.05 was considered statistically significant.

## Competing interests

The authors declare that they have no competing interests.

## Authors’ contributions

LY carried out the construction of HBx plasmids and transfection, participated in the in vitro and in vivo studies and drafted the manuscript. LG carried out the tumor-bearing mice model and participated in the in vivo studies. WW carried out the cell apoptosis and cell cycle analysis by FACS. SY carried out the immunofluorescence and western blot analysis. ZM participated in the design of the study and performed the statistical analysis. CZ conceived of the study, and participated in its design and coordination. All authors read and approved the final manuscript.
